# Pediatric meningioma and seizures: a single-center cohort study

**DOI:** 10.1007/s00381-025-07106-7

**Published:** 2026-01-13

**Authors:** Emma Ye, Drew Hines, Shilpa B. Reddy, Devang J. Pastakia, Michael C. Dewan

**Affiliations:** 1 Vanderbilt University School of Medicine, Nashville, TN USA; 2 Department of Pediatric Neurology, Vanderbilt University Medical Center, Nashville, TN USA; 3 Department of Pediatric Hematology/Oncology, Vanderbilt University Medical Center, Nashville, TN USA; 4 Department of Neurological Surgery, Vanderbilt University Medical Center, Nashville, TN USA

**Keywords:** Pediatric meningioma, Seizures, Gross total resection, Anti-seizure medication, Seizure freedom

## Abstract

**Objective:**

Pediatric meningiomas are rare but often present clinically with seizures. Despite this, seizure outcomes and perioperative seizure management strategies remain underreported in the pediatric population. This paper aims to characterize seizure presentation, evaluate anti-seizure medication (ASM) use, and assess seizure outcomes following surgical resection in children with meningiomas.

**Methods:**

We conducted a retrospective chart review of pediatric patients (< 18 years) who were diagnosed with meningioma at Vanderbilt University Medical Center between 2014 and 2024. For surgically treated patients, the diagnosis was histologically confirmed following resection. For patients managed conservatively, the diagnosis was presumed radiographically based on characteristic dural-based morphology, homogeneous enhancement, and absence of alternative differential considerations on MRI. These presumed lesions demonstrated long-term radiographic stability on serial imaging, supporting the diagnosis of meningioma. Data on seizure presentation, anti-seizure medication (ASM) use, tumor features, extent of resection (EOR), and seizure outcomes were extracted; seizure outcomes were evaluated using the Engel classification.

**Results:**

Sixteen patients were included (median age 15 years), of whom six (37.5%) presented with seizures. Gross total resection (GTR) was achieved in 11 of 12 surgically treated patients (91.7%) and in all six patients (100%) with seizures. All six patients were started on ASMs pre-operatively; however, medication and duration of treatment varied. At a median follow-up of 3.5 years, four patients (66.7%) achieved Engel Class IA outcomes, with two weaned off ASM(s) without seizure recurrence. One patient each (16.7%) was classified as Engel IVC and IVB. Notably, seizures were observed in both patients with neurofibromatosis type 2 (100%), the single patient with radiation-induced meningiomas (100%), and those harboring rare molecular alterations. ASM regimens varied, underscoring the lack of standardized management protocols in this population.

**Conclusions:**

Seizures are a common clinical presentation in pediatric meningioma. While GTR appears beneficial for seizure control, ASM management remains heterogeneous. These findings support the need for consensus-based perioperative seizure management guidelines and further multicenter studies to clarify the relationship between tumor biology, treatment approaches, and long-term neurologic outcomes.

## Introduction

**Fig. 1:**
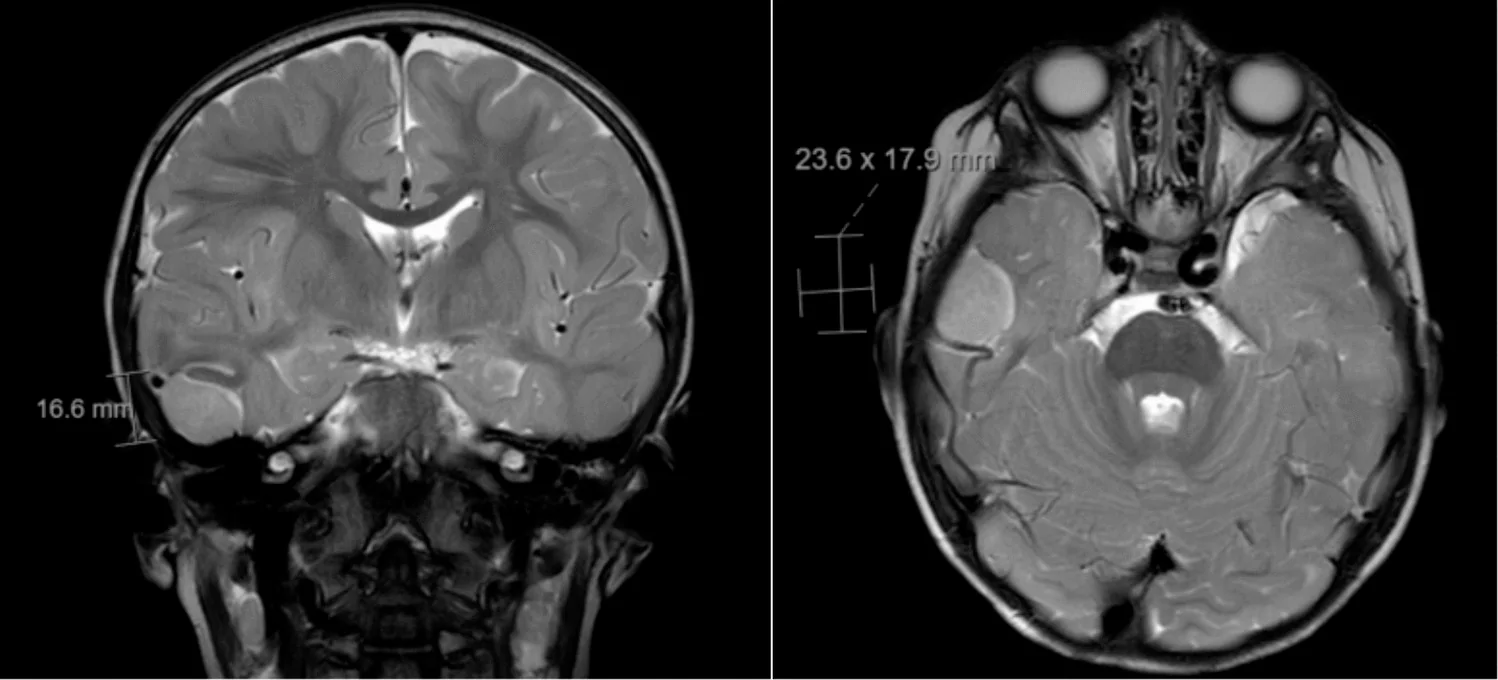
Preoperative MRI of a 16-month-old patient in this cohort demonstrated an extra-axial mass of the right middle cranial fossa with adjacent leptomeningeal enhancement and abnormal signal involving the adjacent right inferior temporal gyrus and sulcus.

Pediatric meningiomas are rare central nervous system (CNS) tumors, accounting for 1–2% of primary intracranial neoplasms in children [1, 2]. They differ biologically and clinically from their adult counterparts, displaying higher rates of atypical or malignant histology, more frequent associations with genetic syndromes such as neurofibromatosis type 2 (NF2), and a greater likelihood of aggressive clinical behavior [3, 4]. Pre-operative MRI images of a pediatric meningioma can be seen in Fig. 1. In addition to tumor recurrence and neurological deficits, seizures represent a common clinically recognized feature of pediatric meningiomas [5, 6]. In adult meningioma populations, seizures are reported in approximately 20–30% of cases, often correlating with tumor location and peritumoral edema [7, 8]. Pediatric studies suggest an even higher seizure prevalence at presentation, with seizure rates ranging from 30 to 50% in some series [2, 5, 9]. However, unlike in adult meningiomas, the factors contributing to seizure development and persistence in children remain poorly characterized. Gross total resection (GTR) has been linked to better outcomes as it improves tumor control and progression-free survival [9, 10]. Several studies in adult populations have suggested that GTR may also improve seizure outcomes, yet this relationship has been minimally studied in pediatric cohorts [8]. Similarly, the role of anti-seizure medications (ASMs) in the perioperative management of meningioma-associated seizures is not well-defined in children. Pediatric brain tumor guidelines do not provide consensus recommendations on seizure prophylaxis or post-operative management, leading to variability in clinical practice [2].

Critical questions regarding the role of tumor grade, histologic subtype, molecular alterations, and extent of resection (EOR) in seizure pathogenesis and outcomes remain unanswered. This study aims to (1) characterize the frequency of seizure presentation in pediatric meningioma, (2) describe seizure outcomes following surgical resection, (3) evaluate the impact of EOR on seizure control, and (4) examine patterns of ASM use and their relationship to seizure freedom. By focusing on seizure and ASM outcomes, this study explores clinical domains largely absent from prior pediatric meningioma series and provides new insights into the management of seizures in this rare patient population.

## Methods

A retrospective chart review was conducted of all pediatric patients (age < 18 years) who were diagnosed with meningioma at our institution between 2014 and 2024. For surgically treated patients, the diagnosis was histologically confirmed following resection. For patients managed conservatively, the diagnosis was presumed radiographically based on characteristic dural-based morphology, homogeneous enhancement, and absence of alternative differential considerations on MRI. These presumed lesions demonstrated long-term radiographic stability on serial imaging, supporting the diagnosis of meningioma (median imaging follow-up duration: 3.4 years). Data were extracted on demographics, seizure presentation, seizure outcomes, ASM use (pre-operative, post-operative, and at follow-up), tumor location, WHO grade, and extent of resection. EOR was classified based on operative and imaging reports, with GTR defined as complete resection and subtotal resection (STR) including partial or undocumented resections [9]. Seizure outcomes were assessed through clinical follow-up and categorized using the Engel Epilepsy Surgery Outcome Scale to allow standardized comparison of seizure control [11]. ASM regimens were reviewed to evaluate patterns of initiation, continuation, and discontinuation.

## Results

### Sex and age

A total of 16 pediatric patients with meningioma were included in this study. The median age at diagnosis was 15 years (interquartile range [IQR]: 11.4–16.5 years), and the cohort consisted of 7 females (43.8%) and 9 males (56.3%). The male-to-female ratio was 1.3:1. Their clinical courses were summarized in Table 1.

**Table 1:** Patient abstract

Patient number	Age at surgery	Sex	Presenting symptoms	Location	Extent of resection	WHO Grade	Tumor Histology	Molecular characteristic	Previous radiotherapy or associate genetic condition	Recurrence	Number of procedures	Adjuvant therapy	ASM before surgery	ASM after surgery	Seizure freedom achieved	Follow-up duration (Month)	Engle Class
1	17	M	H; S	Anterior falcine	GTR	I	Meningothelial	-		N	1	N	Y	Y	Y	57	IA
2	13	M	S	L occipital lobe	GTR	II	Atypical	15q duplication	15q duplication syndrome	N	1	N	Y	Y	N (seizure free for 18 months)	44	IC
3	15	F	H	Anterior inferior parietal lobe	GTR	II	Atypical	-		N	1	N	-	-	-	39	-
4	16	F	S	R frontotemporal lobe	GTR	II	Atypical	-	Previous radiotherapy for ALL	N	1	N	Y	Y	N	32	IVB
5	1.3	M	S	R temporal	GTR	II	Atypical	YAP1::LMO1 Rearrangement		N	1	N	Y	Y	Y (On ASM)	8	IA
6	3	M	A	L frontal	GTR	-	-	-		N	1	N	-	-	-	5	-
7	12	F	A	R parafalcine	-	_	-	-	-	N	1	N	-	-	-	11	-
8	16	M	W	L parafalcine	GTR	I	Meningothelial	-		N	1	N	-	-	-	57	-
9	16	M	S	L frontal lobe	GTR	I	Meningothelial	NF2	NF2	N	1	N	Y	Y (Weaned after 1 year)	Y	46	IA
10	16	F	S; H	Bilateral frontal	GTR	II	Atypical	NF2	NF2	Yx3	4	Y (Radiation therapy)	-	Y (Weaned after 1 year)	Y	23	IA
11	6	F	V	R optic nerve	STR	I	Meningothelial	-	-	N	1	Y (Radiation therapy)	-	-	-	165	-
12	16	M	A	Frontal parafalcine	-	-	-	-	-	N	1	N	-	-	-	53	-
13	6	M	V	R optic nerve	GTR	I	Meningothelial	-	-	N	1	N	-	-	-	68	-
14	16	F	A	L lateral ventricle	GTR	I	Transitional	-	-	N	0	N	-	-	-	-	-
15	13	F	A	Anterior falcine	-	-	-	-	-	N	0	N	-	-	-	29	-
16	15	M	A	R Frontal	-	-	-	-	-	-	0	-	-	-	-	67	-

*H* headache, *S* seizure, *A* asymptomatic, *W* weakness, *V* visual disturbances, *GTR* gros total resection, *STR* subtotal resection, *NF2* neurofibromatosis type 2, ASM antiseizure medication, “- “ Not Available

### Presenting symptoms and signs

Of the 16 patients, 6 (37.5%) patients were asymptomatic at presentation. Among the 10 patients with reported symptoms, seizures were the most common presenting symptom (6 patients, 37.5%), followed by headaches (3 patients, 18.8%), visual disturbances (2 patients, 12.5%), and focal weakness (1 patient, 6.3%).

### Tumor location

Tumor locations were distributed across several intracranial regions. Five cases (31.3%) were located in the falcine region, and four (25%) in the frontal lobes. Two cases (12.5%) involved the optic nerve. One case each (6.3%) was located in the occipital lobe, parietal lobe, temporal lobe, lateral ventricle, and frontotemporal lobe.

### Extent of resection and seizure outcome

Of the 16 patients, 11 (68.8%) underwent gross total resection (GTR), one (6.3%) underwent subtotal resection (STR) for an optic nerve meningioma, and four (25%) were managed conservatively. All six (100%) patients who presented with seizures underwent GTR. Five (83.3%) achieved seizure freedom at one-year follow-up and four patients (66.7%) remained seizure-free at 3.5-year follow-up. To standardize outcome reporting, we applied the Engel classification, finding that four patients (66.7%) achieved Class IA outcome (complete seizure freedom). One patient (16.7%) was categorized as Class IVC, and another (16.7%) as Class IVB.

### Histopathologic subtype and seizure

Histopathologic analysis revealed WHO Grade I meningiomas in six patients (37.5%) and WHO Grade II (atypical) meningiomas in five patients (31.3%). In one surgically treated patient, the specimen was too small to be formally graded by WHO criteria; however, no atypical features were identified, and the proliferation index (Ki-67) was low, consistent with a low-grade neoplasm. Among patients with known histologic subtypes, five patients (45.5%) had meningothelial subtypes and five (45.5%) had atypical subtypes, while one patient (9.1%) had a transitional meningioma. Seizures occurred in four of five patients (80%) with Grade II tumors, and in two of seven patients (28.6%) with Grade I tumors.

### Radiation-induced meningioma and seizure

One patient (6.3%) in our cohort was diagnosed with radiation-induced meningioma (RiM). This patient had a prior history of acute lymphoblastic leukemia (ALL) treated with chemotherapy and total body irradiation during infancy. This patient presented with seizures and continued to experience breakthrough epileptic episodes on postoperative ASM therapy.

### Molecular features and neurofibromatosis status

Two patients (12.5%) had a documented diagnosis of NF2 and both (100%) presented with seizures. In addition, molecular alterations were identified in two other patients: one with a 15q duplication and another with somatic YAP1:LMO1 gene rearrangement. Both patients presented with seizures.

### Adjuvant therapy

Adjuvant therapy was utilized in a limited subset of patients. Two patients (12.5%) received post-operative radiation therapy. One of these patients had a WHO Grade II tumor and presented with seizures; this patient experienced multiple recurrences, had a confirmed diagnosis of NF2, and underwent adjuvant radiation, receiving a total dose of 59.4 Gy in 33 fractions. The other patient underwent subtotal resection for a WHO Grade I optic nerve meningioma and received adjuvant radiation due to limited surgical accessibility, with a total dose of 54.0 Gy.

### ASM for pediatric meningioma

All six (100%) patients who presented with seizures were initiated on ASM(s) pre-operatively (median duration six months prior to meningioma resection) and remained on ASM(s) post-operatively (median duration 18 months). Among these, two patients received polytherapy (lamotrigine, diazepam, and zonisamide), two were on levetiracetam monotherapy, and one received a combination of clonazepam, perampanel, and cenobamate. Median follow-up interval was 3.5 years. Five patients (83.3%) achieved seizure freedom at one-year follow-up and four patients (66.7%) at 3.5-year follow-up. Of these four patients, two were successfully weaned off one year after starting ASM, while one remained on ASM. The remaining two patients had persistent seizures, including one who initially achieved seizure freedom for 18 months before epilepsy recurrence.

### Recurrence and re-intervention

Tumor recurrence occurred in one patient (6.3%) with a WHO Grade II (atypical) meningioma and a diagnosis of NF2. This patient initially presented with seizures and underwent gross total resection. Despite aggressive treatment, the tumor recurred locally twice at an outside hospital, at intervals of approximately two and three years, before a third recurrence was treated at our institution at an interval of five years. At our center, she underwent surgical resection with adjuvant therapy and was followed for 12 months, during which she remained seizure-free after being weaned off ASMs. No other cases of recurrence were observed in the cohort.

### Case highlight: infant meningioma (16-month-old patient)

**Fig. 2:**
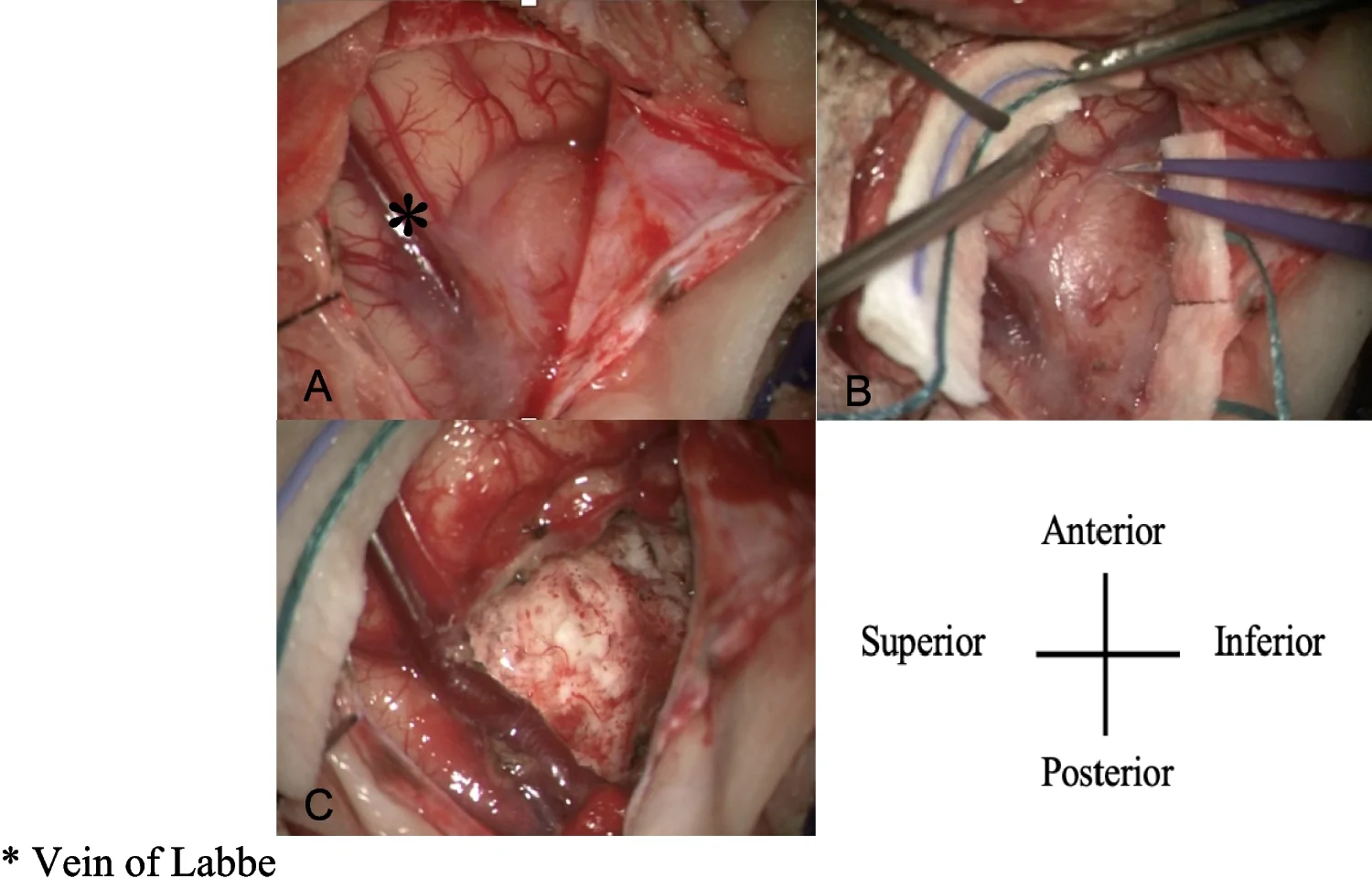
Intraoperative images of the right temporal exposure as viewed through the operating microscope. Patient anterior is oriented at the top of the screen and superior to the left. The extra-axial lesion is seen extending around and partially draping over the vein of Labbe (**A**, **B**) as it courses inferiorly and posteriorly. Following complete microsurgical excision, no residual tumor is visualized, and the vein of Labbé is preserved and fully exposed along its length (**C**). *Vein of Labbe

A 16-month-old male presented with new-onset focal seizures progressing to status epilepticus. He had been previously healthy with no history of trauma, infection, developmental delay, or family history of epilepsy. Initial CT performed at an outside hospital demonstrated a small right temporal hyperdensity. MRI obtained on transfer to our institution revealed an extra-axial mass along the floor of the right middle cranial fossa with adjacent leptomeningeal enhancement and abnormal cortical signal involving the right inferior temporal gyrus (Fig. 1). The patient underwent right temporal craniotomy, and gross total resection was achieved (Fig. 2). Pathologic evaluation demonstrated an atypical meningioma (WHO Grade II) with mitotic activity and multifocal necrosis. Molecular testing identified a YAP1::LMO1 rearrangement, with no TERT promoter mutation or CDKN2A/B deletion. The postoperative course was uncomplicated. At two-week and eight-month follow-up visits, he remained seizure-free on ASM, with no new neurologic deficits.

## Discussion

Pediatric meningiomas are rare but clinically significant tumors, often presenting with neurological deficits, most commonly, seizures. In our single-center cohort, seizures were a presenting symptom in 37.5% of patients, consistent with prior literature reporting seizure rates between 30–50% in pediatric meningioma populations [5, 9]. Though pediatric meningioma can occur throughout childhood, the typical age at presentation ranges from 10 to 16 years; the median age in our cohort was 15 years [2]. Unlike adult meningiomas, which show a clear female predominance thought to be influenced by hormonal effects on meningioma cell receptors [2, 3, 12], our cohort demonstrated a male-to-female ratio of 1.3:1. This pattern aligns with prior pediatric studies reporting a slight male predominance, suggesting that the pathogenic mechanisms driving tumor development in children may differ from those in adults [5, 10].

Surgical resection has been the treatment of choice for meningioma [8, 10]. Prior literature in adult populations has linked extent of resection to improved seizure outcomes [13], but data in the pediatric setting remain limited with only a few studies suggesting higher rates of postoperative seizure freedom following GTR [8, 14]. Twelve out of 16 patients in our cohort underwent surgical treatment. GTR was performed in 11 patients (91.6%), including all six patients who presented with seizures. Among these six patients, five (83.3%) achieved seizure freedom at one-year follow-up and four remained seizure-free at 3.5-year follow-up, two of whom were weaned off ASM(s). To standardize outcome reporting, we applied the Engel classification at 3.5-year follow-up [11]: four patients (66.7%) achieved Class IA, indicating complete seizure freedom; one was classified as Class IVC, reflecting worthwhile improvement despite persistent seizures; and another as Class IVB, indicating no worthwhile improvement. Given these heterogeneous seizure outcomes, we further examined the two patients who continued to experience postoperative seizures to better characterize potential contributors to persistent epilepsy. Both patients underwent comprehensive postoperative epilepsy evaluations, highlighting the complexity of seizure persistence even after adequate tumor control. In one patient, epileptic events were infrequent and typically occurred during intercurrent illness; postoperative EEGs showed no epileptiform activity, and MRI demonstrated only expected postsurgical changes without recurrence, with a small indeterminate enhancing focus remaining under surveillance. In contrast, the second patient experienced worsening postoperative seizures. Continuous EEG monitoring revealed focal impaired-awareness and generalized seizures lateralized to the right hemisphere, and PET imaging demonstrated right temporal and parietal hypometabolism. Follow-up MRI showed stable surgical changes with mild adjacent dural enhancement. This patient was subsequently evaluated by the epilepsy surgery team and formally recommended for an anterior two-thirds corpus callosotomy. These cases underscore that seizure persistence in pediatric meningioma may reflect underlying cortical irritability or broader epileptogenic networks rather than residual tumor alone [15]. They also highlight the importance of postoperative neurophysiologic assessment and referral for advanced epilepsy management when seizures continue despite tumor resection.

While limited by small sample size, these observations are consistent with the hypothesis that maximal safe resection may play a pivotal role in optimizing seizure outcomes in children with meningioma [9–11]. Larger, multi-institutional pediatric cohorts will be necessary to determine whether resection extent independently influences seizure outcomes when accounting for tumor location, histologic subtype, and molecular characteristics. Beyond the role of resection, postoperative seizure control is also shaped by medical management. Despite the frequent occurrence of seizures in pediatric meningioma, seizure management and outcomes, particularly regarding ASM use, remain underreported in the literature [8, 9, 15]. In our cohort, all six patients who presented with seizures received ASM therapy. The choice of ASM varied, with agents including levetiracetam, lamotrigine, diazepam, zonisamide, clonazepam, perampanel, and cenobamate used in different combinations. In our retrospective review, ASM selection was dependent on physician preference and individualized clinical judgment, with no standardized institutional protocol guiding medication choice, initiation, or tapering. This variability reflects the individualized nature of ASM prescribing in pediatric practice and the limited data available to inform standardized approaches in this setting.

Molecular and genetic alterations are increasingly recognized as important factors in pediatric meningioma pathophysiology, but their association with seizure risk remains poorly defined [8]. In our cohort, two (12.5%) patients were diagnosed with NF2 and both presented with seizures, raising the possibility that NF2-related tumor biology may be associated with epileptogenesis [10]. Although the mechanisms remain unclear, prior reports suggest that multifocal tumor growth, peritumoral edema, and cortical irritation may contribute to higher seizure burden in NF2 patients. In addition to NF2, two other patients in our cohort harbored notable molecular alterations; one with a somatic 15q duplication and another with a YAP1:LMO1 gene fusion, both of whom presented with seizures [3, 10]. While these alterations have been described, their contribution to seizure development is not well understood. Most tumors in our cohort were WHO Grade I or II, and no definitive associations between seizure presentation and tumor grade, histology, or molecular profile could be established given the small sample size. These findings highlight the need for larger molecularly annotated pediatric cohorts to better determine the genetic drivers of seizure risk and to inform precision management strategies.

Meningiomas in infants and toddlers are exceedingly uncommon, and the presence of a YAP1::LMO1-rearranged atypical meningioma in a 16-month-old patient in our cohort further illustrates the distinctive clinical and molecular features seen in this age group. YAP1-fusion–positive, NF2-wild-type meningiomas represent a unique pediatric subset with biology that differs from the NF2-driven tumors more commonly observed in older children and adults [16, 17].

Radiation-induced meningioma (RiM) is an uncommon but well-recognized long-term complication of cranial or total body irradiation, typically developing after a latency period of more than a decade [16, 17]. In our cohort, one patient developed a WHO Grade II meningioma approximately 10 years after receiving chemotherapy and total body irradiation for acute lymphoblastic leukemia (ALL). The diagnosis of RiM was established according to Cahan’s criteria, with no evidence of genetic predisposition or alternative etiologies [18]. The patient presented with seizures and continued to experience breakthrough episodes despite postoperative ASM therapy. While the mechanisms linking radiation exposure to epileptogenesis remain unclear, prior studies suggest that cortical scarring, vascular injury, and peritumoral gliosis may contribute to lowering the seizure threshold [8]. Data on seizure outcomes in pediatric RiM are extremely limited, underscoring the need for further study of this rare clinical entity.

Our study has several limitations. As a retrospective, single-center analysis, it is inherently subject to selection and information biases. The small sample size limits the power to detect statistically significant associations, and seizure outcomes were largely determined by chart review, with follow-up duration varying across patients. Moreover, ASM prescribing practices were not standardized and reflected individual provider preferences rather than a protocolized approach, making it difficult to draw definitive conclusions about optimal peri-operative seizure management. Despite these limitations, this study addresses a key gap in the pediatric meningioma literature by providing data on seizure management and outcomes in our cohort. Our findings underscore the limited evidence available to guide post-operative seizure management in children with meningioma. Although treatment generally follows standard principles for lesion-related epilepsy, there remains little pediatric-specific data to inform decisions around ASM initiation, duration, and tapering in this population. Prospective multicenter studies are needed to clarify optimal management strategies and to validate the trends observed in our cohort. In addition, given the distinct molecular features of pediatric meningiomas compared with adult disease, further investigation into the biological underpinnings of epileptogenesis in this group may help refine risk stratification and support more individualized care.

## Conclusion

Seizures are a clinically recognized manifestation of pediatric meningiomas, yet their management and long-term outcomes remain poorly defined in the literature. This single-center retrospective study provides new insights into seizure management, ASM use, and post-surgical outcomes in a contemporary pediatric cohort. The inclusion of molecular data and Engel seizure classification adds granularity to the understanding of seizure outcomes. Given the rarity of pediatric meningioma and the limited available data on seizure outcomes, larger prospective, multicenter studies are essential. These efforts will be critical in guiding clinical decision-making, developing consensus-based ASM management strategies, and improving quality of life for this vulnerable patient population.

## Data Availability

No datasets were generated or analysed during the current study.
